# Evaluation of potential sources of nosocomial infection in endodontic practice: a hygienic study

**DOI:** 10.1038/s41405-025-00378-w

**Published:** 2025-12-12

**Authors:** Stanislav Voroshilov, Baghish Harutyunyan, Vigen Goginyan, Michael Solomonov

**Affiliations:** 1 Laboratory of Energy Alternative Sources, Scientific and Production Center “Armbiotechnology” of the National Academy of Sciences, Yerevan, Armenia; 2 Department of Endodontics, Israel Defense Forces (IDF) Medical Corps, Tel Hashomer, Israel; 3 “Bina” Program, Faculty of Dental Medicine, Hebrew University of Jerusalem, Jerusalem, Israel

**Keywords:** Endodontics, Root canal treatment

## Abstract

**Aim:**

This hygienic study assesses the microbial contamination of objects commonly used in a clinic during routine endodontic procedures.

**Methods:**

Samples were collected from clinical surfaces and instruments. Microbial colonies were cultured on agar, enumerated, and identified phenotypically, including assessment of antibiotic resistance.

**Results and conclusions:**

Findings revealed that the majority of objects in routine endodontic practice are predominantly contaminated by skin-derived *Staphylococcus epidermidis* and *Micrococcus luteus*, which frequently exhibit antibiotic resistance, whilst *Stenotrophomonas maltophilia* is also a frequent contaminant, particularly in DUWL. Other items, including rubber dam sheets, gloves, paper points, and paper mixing pads, showed detectable microbial contamination. These results highlight the presence of potentially nosocomial microorganisms on routine endodontic surfaces, emphasising the importance of appropriate hygiene measures in endodontic practice.

## Introduction

Apical periodontitis is an infectious disease, and the primary goal of endodontic treatment is high-quality disinfection [1, 2]. This is achieved during the chemo-mechanical preparation of the dental root canal, which reduces the microbial load to a level that allows the lesion to heal [1, 2]. Success rates for such treatment range between 75 and 85%, depending on whether it is a primary treatment or a retreatment [1, 2]. Achieving the complete elimination of microorganisms from all areas of the root canal system—such as isthmuses and other unprepared parts of the canal—is often impossible [1, 2].

During the endodontic treatment of vital teeth, the goal is to prevent apical periodontitis. In these cases, the success rate appears to be 95% when assessed via 2D X-rays [3]. However, studies utilising the more sensitive 3D cone-beam computed tomography (CBCT), such as those conducted by Patel et al. [4], revealed a success rate closer to 83%. This relatively high percentage of post-treatment apical periodontitis may be partially attributed to the introduction of nosocomial infections during the procedure.

Despite rigorous infection-control protocols, achieving and maintaining a truly aseptic field during endodontic treatment remains challenging. Recent evidence shows that even with strict measures in place—such as rubber dam isolation, instrument replacement, and field disinfection—bacterial contamination can still occur. These findings underscore the clinical urgency of identifying and mitigating potential nosocomial sources of contamination in endodontic practice [5, 6].

Additionally, nosocomial infections might influence cases with pre-existing infections by interacting with the existing microorganisms, potentially complicating the infection dynamics and treatment outcomes. Furthermore, nosocomial contamination during endodontic procedures may lead to transient bacteraemia, potentially affecting systemic health. This is particularly relevant for medically compromised patients, where such bacterial entry may increase the risk of conditions like infective endocarditis [7].

By identifying the sources and types of specific nosocomial infections, we can develop a better understanding and protocols to prevent their occurrence, which may influence the overall success rates of endodontic treatments.

The purpose of our study was to assess the bacterial contamination present on various objects commonly utilised in routine endodontic procedures during patient treatment. It should be acknowledged that strict comparison of microbial loads per surface unit is not feasible due to differences in items’ shapes.

## Methodology

### Sampling and culturing

This study was conducted in a single private dental setting using the same dental unit during endodontic treatment, which ensured consistency in clinical protocols and sampling procedures. However, this limits the generalisability of the findings, as microbial contamination levels may vary across clinics depending on patient populations, staff, and specific aseptic routines.

Eight potential sources of nosocomial bacteria were targeted:

- mixing pads;
- paper points;
- water from a dental unit water line (DUWL);
- gutta-percha points;
- rubber dam sheets (work and near-the-nose parts);
- gloves (work and near-the-skin parts);
- endodontic dispensers (reusable syringes for irrigation);
- endodontic files (stored in an open sponge after sterilisation).

For each clinical source, nine samples were collected immediately prior to root canal obturation. Patients varied in age and sex. Sampling was distributed across different days of the week, typically 4–5 times per week, and at various times during clinical hours to ensure representative coverage and reduce temporal bias. All the essential information on sampling and culturing is summarised in Table 1.

**Table 1:** Experimental framework used in the study.

	Workflow steps				
	Collecting	Transporting to laboratory	Seeding to the agar	Incubation	Analysis of the obtained colonies
Paper mixing pads	Collected a sample from 1 pad with a cotton swab moisturised in PBS	The samples were transported to the laboratory within 1–2 h at RT	For all samples, the seeding was performed after vortexing (20 s for samples obtained from instruments, and 10 s for others), then transferring 300 μl of a sample to the agar, followed by spreading with a Drigalski spatula. Additionally, to ensure that all anaerobes are found, the seeding was performed by transferring samples from the gel with a cotton swab, followed by spreading with the swab.	For aerobes, the incubation was performed in a thermostat at 37° C for 3 days. For anaerobes, the incubation was performed in a GENbox anaer® anaerobic chamber for 7 days at 37 °C.	The number of bacteria was evaluated via colony counting. Microscopic identification was performed for all samples. For aerobes, species were identified phenotypically via the specific enzyme activity in Render MA 120^TM^, utilising 24 substrates. For anaerobes, species were identified phenotypically by the specific enzyme activity in Vitek 2®, utilising an ANC card with 36 substrates.
Paper points	Put 5 paper points in a tube				
DUWL	Poured approx. 1 ml of water from a DUWL into an empty tube				
Gutta-percha points	Put 3 gutta-percha points in a tube filled with PBS				
Rubber dam sheets (work and near-the-nose parts)	Collected a sample with a cotton swab, moisturised in PBS				
Gloves (work and near-the-skin parts)	Collected a sample with a cotton swab, moisturised in PBS				
Endo dispensers	Collected a sample with a cotton swab, moisturised in PBS				
Endo files H-files, K-files + K-like rotary	Put 3 files in a tube filled with PBS				

The time elapsed between the sample collection and culturing was ~1–2 h. All samples were transferred into 4 ml transport tubes for clinical microbiological analyses, each tube filled with 1 ml of phosphate-buffered saline (PBS). Heat-insulating bags were used to maintain an environment close to room temperature (~20 °C). Swabs from surfaces such as rubber dam sheets, mixing pads, dispensers, and gloves were collected using a cotton tip moistened with sterile PBS. The swabs were meticulously rubbed against most parts of the surfaces, immersed in PBS-filled tubes, and well shaken.

For consistency, entire surfaces of standardised items were sampled. Samples from rubber dam sheets (152 × 152 mm) and mixing pads (60 × 60 mm) were collected by thorough wiping with sterile PBS-moistened cotton swabs. We emphasize the clinical relevance of total microbial load per item over microbial load per cm² due to the heterogeneity in size and shape of sampled items.

Samples from files, gutta-percha points, and paper points were collected by immersing the items into tubes filled with PBS.

The blank control samples with just sterile PBS served as a negative control for aseptic technique verification. The total study sample was 12 × 9 = 108 samples from presumably contaminated sources plus control of sterility.

An amount of 300 μl of each sample was transferred with a pipette to agar plates and spread using a Drigalski spatula. To identify aerobes, the plates were incubated aerobically in 90 mm Petri dishes with Columbia agar containing 5% of sheep blood (Liofilchem®) for 3 days. Anaerobic samples were incubated in 90 mm Petri dishes with Schaedler agar containing 5% of sheep blood (Liofilchem®) in a GENbox anaer® (bioMérieux™) anaerobic chamber for 7 days. All samples were incubated at 37 °C.

For samples without solid items transported in PBS, 10 s of vortexing in transport tubes was performed, followed by transferring the samples to the agar. Samples with instruments, gutta-percha points, and paper points underwent 20 s of vortexing, and were then transferred with a pipette to the agar.

Antibiotic sensitivity tests were performed using antibiotic discs (Liofilchem®) on agar, followed by determination of the zone of growth inhibition according to the EUCAST guidance of 2023 [8].

The antibiotics used in the test were Azithromycin, Trimethoprim, Clarithromycin, Penicillin, Oxacillin, Erythromycin, Linezolid, Tetracycline, Rifampin, Minocycline, Vancomycin, Methicillin, Ceftriaxone, Daptomycin, Doxycycline, Chloramphenicol, Ciprofloxacin, Levofloxacin, Moxifloxacin, Gentamicin, Tobramycin, Amikacin, Teicoplanin, Tigecycline, Ampicillin, Streptomycin, Meropenem, Imipenem, Ceftazidime, Piperacillin, Cefepime, Cefotaxime, Polymyxin B, Ticarcillin, Colistin, Sulbactam, Aztreonam, Cefuroxime, Amoxiclav, Clindamycin, and Metronidazole.

### Microbiological analysis

After incubation, colonies were counted and analysed by visual, microscopic, and phenotypic methods through the specific enzyme activity tests. Aerobes were identified in Render MA 120^TM^ (Render Biotech Co., LTD.), utilising 24 substrates for species identification. Anaerobes were identified in VITEK^®^ 2 with ANC cards (bioMérieux™), utilising 36 substrates for species identification. It should be acknowledged that phenotypic identification used in this research detects only culturable bacteria and may have lower resolution than genotypic approaches such as 16S rRNA gene sequencing, which can profile both culturable and non-culturable organisms. Additionally, antibiotic susceptibility tests were conducted on the obtained strains.

### Data analysis

Data visualisation was performed using Python (Matplotlib library). The graphs represent the raw counts and frequencies of microbial species across all samples and clinical sources. No statistical analyses were conducted; the figures present descriptive trends in species prevalence and CFU counts.

## Results

### Species composition of contaminants in the endodontic environment

**Fig. 1:**
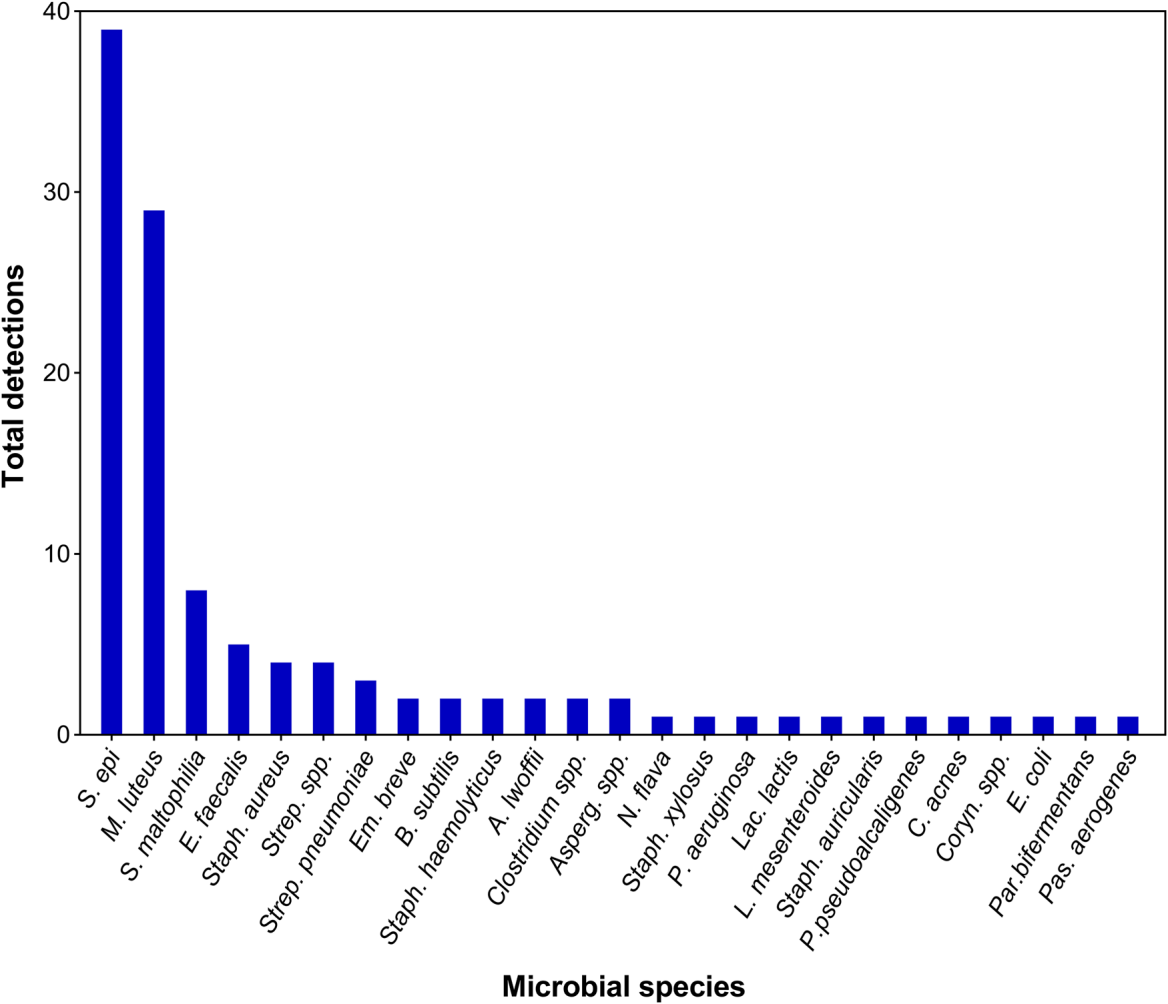
Total detections of microbial species across all samples of all clinical sources. X-axis represents the microbial species identified across all endodontic sources. Y-axis represents the number of detections, i.e., how many times each species was recovered across all collected samples.

**Fig. 2:**
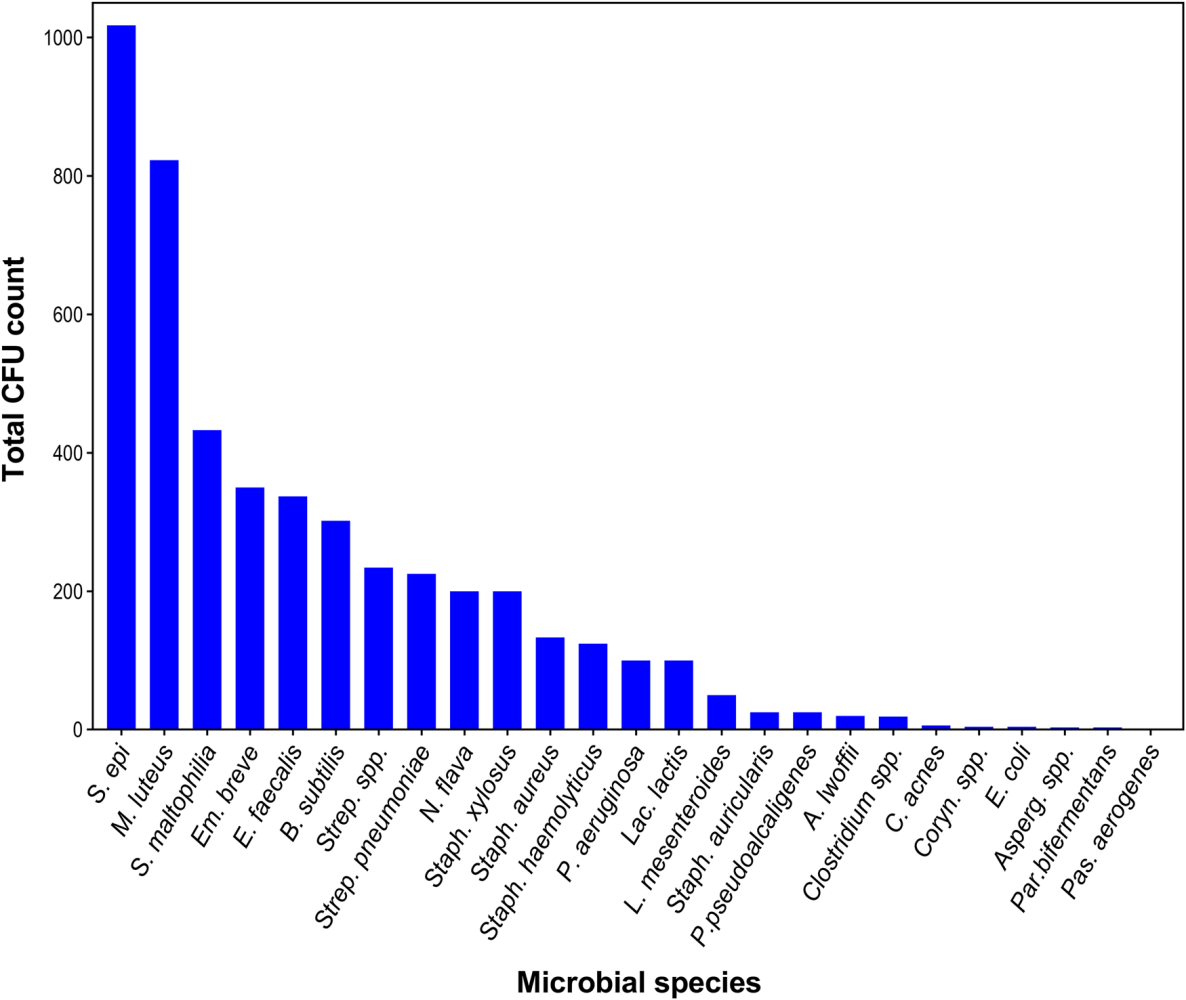
Total CFU counts of microbial species across all samples of all clinical sources. X-axis represents the microbial species identified across all endodontic sources. Y-axis represents the total colony-forming unit (CFU) counts accumulated for each species across all samples. This figure illustrates the relative microbial load contributed by each detected species. It should be noted that the data are presented descriptively and do not imply direct statistical comparison between species.

Across all sources, 19 microbial genera and 21 species were identified in samples from objects employed in routine endodontic practices using culture-based methods. Contamination was dominated by *Staphylococcus epidermidis* (39 detections, 1018 CFU) and *Micrococcus luteus* (29 detections, 823 CFU). Other species were detected less frequently, including *Stenotrophomonas maltophilia*, *Enterococcus faecalis*, *Empedobacter brevis*, *Bacillus subtilis*, *Streptococcus pneumoniae*, *Neisseria flava*, and *Staphylococcus xylosus*, each recovered fewer than 10 times with total CFU counts below 500. Several additional taxa were isolated only sporadically [Figs. 1, 2].

### Species diversity of each endodontic source

**Fig. 3:**
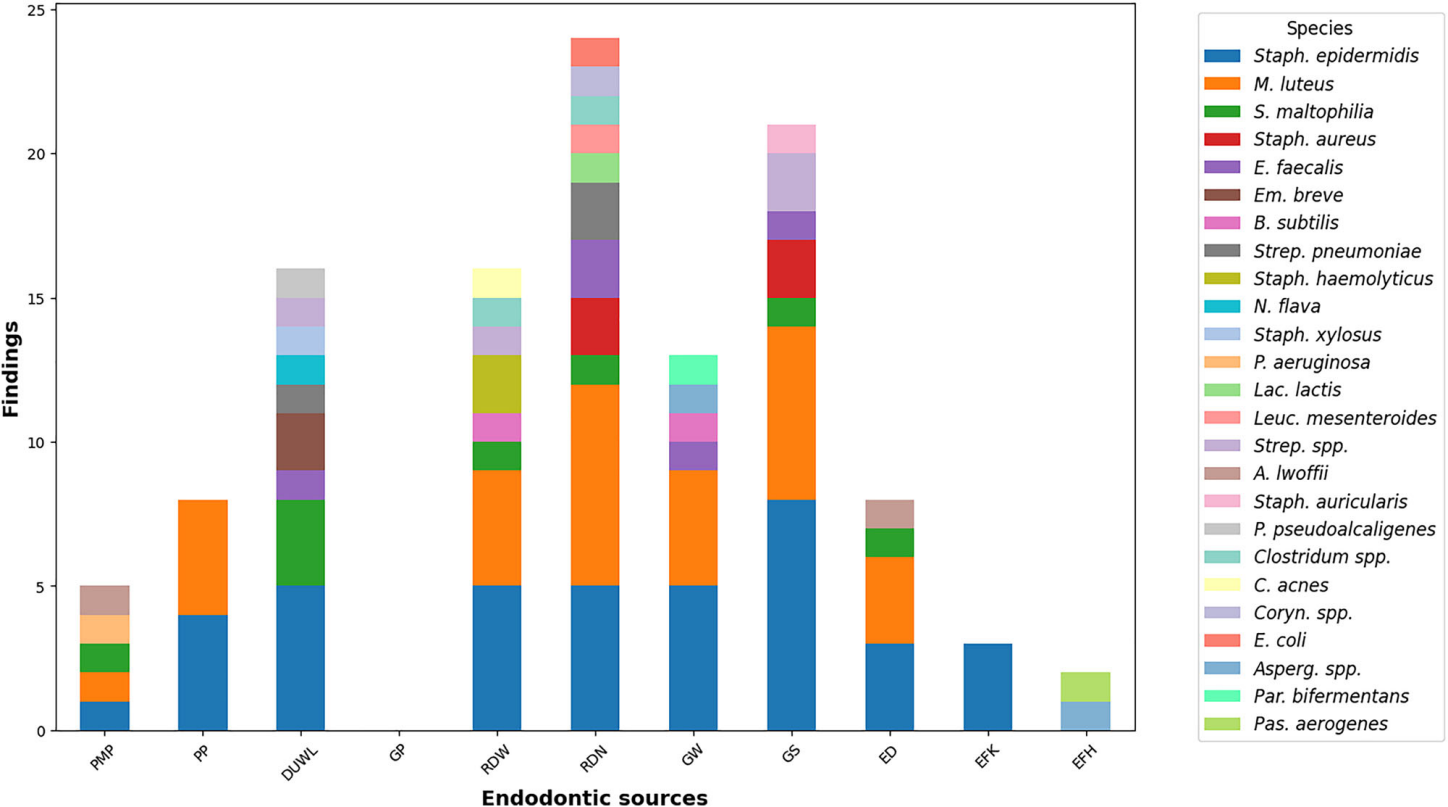
Species diversity of each endodontic source. Species identities are denoted in each column, sorted by the total number of findings (vertical axis). The more frequently a species is found, the longer is the segment representing that species in a column. The tested materials (horizontal axis) were PMP (paper mixing pads), PP (paper points), DUWL (dental unit water line), GP (gutta-percha points), RDW (working region of rubber dam sheets), RDN (near-the-nose region of rubber dam sheets), GW (working region of gloves), GS (near-the-skin region of gloves), ED (endodontic dispensers), EFK (endodontic K-files), and EFH (endodontic H-files).

The distribution of microbial species varied across clinical materials and surfaces Fig. 3.

*Micrococcus luteus* and *Staphylococcus epidermidis* were predominant in almost all sources.

The near-the-nose area of rubber dam sheets harboured the most diverse community (11 species), with *S. epidermidis* and *M. luteus* present in approximately half of the samples.

Dental unit waterlines (DUWL) contained nine species and were notable for the frequent recovery of *S. maltophilia* alongside *S. epidermidis*.

The working area of rubber dam sheets (8 species), the near-the-skin region of gloves (7 species), and the working region of gloves (6 species) also showed high levels of diversity.

Other clinical items, such as paper points, endodontic files, and dispensers, yielded a narrower range of microorganisms, whilst gutta-percha points showed no detectable contamination.

### Antibiotic resistance of endodontic contaminants

Antibiotic susceptibility testing was feasible for the two most common species, *S. epidermidis* and *M. luteus*. Tests were also performed for *S. aureus*. All demonstrated resistance to multiple antibiotics, see Supplementary Table S1.

## Discussion

Apical periodontitis is an infectious disease, and its healing depends on the quality of the dental root canal disinfection during endodontic procedures [1, 2]. The emergence of apical periodontitis after the treatment of vital, non-infected teeth has been reported in 5–17% of cases [3, 4]. This raises questions about how bacteria could contaminate the root canal during the endodontic treatment, especially when a rubber dam is routinely used. One possible route discussed in the literature is nosocomial infection [9]. According to modern infection control concepts, all endodontic instruments, surfaces, and materials involved in the treatment must be sterile [10, 11]. However, in real clinical situations, aseptic control does not achieve ideal sterility [9, 10, 12–15].

Several factors contribute to this challenge. For example, some endodontic items, such as mixing pads, paper points, and gutta-percha points, are challenging to sterilise. Additionally, both personnel and patient skin carry a significant amount of microbiota, creating a microbial presence around the patient throughout their time in the clinic.

Previous studies have examined microbial contamination of selected endodontic items, such as rubber dams and mixing pads, or explored the relation of nosocomial bacteria with bacteraemia. Our findings complement these reports by extending the analysis to a broader set of routinely used clinical materials within one practice setting [9, 10, 12].

From the perspective of bacterial species prevalence, we observed that most species were skin-derived bacteria, with *Micrococcus luteus* and *Staphylococcus epidermidis* frequently dominating. This observation supports the assumption that nosocomial microbiota prevalent in endodontic practice are skin-derived, and this is consistent with data from other studies [9, 16]. However, unlike some other studies that frequently identified *Cutibacterium acnes* [9, 16], our study detected this species only once. This discrepancy may be due to a lower prevalence of *Cutibacterium acnes* carriers compared to the student clinic referenced in this research [9]. The prevalence of specific bacteria is likely influenced by the type of clinic; for example, a student clinic with predominantly young personnel and patients might yield different results compared to a single-doctor clinic with mature patients.

A significant difference in species composition was detected in samples from DUWL, with frequent occurrences of *Stenotrophomonas maltophilia*. It appears that all of the tested species, except for *Bacillus subtilis*, are equally opportunistic pathogens, and none of them poses a greater threat than the others. We assume that the primary risk factors include antibiotic resistance (see Table S1), bacterial load, and the ease of insertion into the root canal or periapical tissues.

Recent evidence has also highlighted the systemic relevance of endodontic microbiota. For example, Gomes et al. (2023) demonstrated that microorganisms present in combined endodontic–periodontal infections may contribute to the risk of endocarditis [17]. Similarly, Niazi et al. (2010, 2025) showed that opportunistic pathogens such as *Staphylococcus epidermidis* and *Cutibacterium acnes*, frequently isolated from refractory endodontic lesions, are capable of systemic dissemination and should therefore be regarded as clinically significant [7, 16, 18]. These findings reinforce the importance of recognising nosocomial contamination not only as a local endodontic concern but also as a potential contributor to systemic disease.

Below is an overview of frequently found bacterial species encountered, highlighting their relationship with infections.

*Staphylococcus epidermidis* may cause periodontitis [16, 19] and is a common cause of implant-associated infections [20]. This bacterium, along with *C. acnes*, plays a significant role in skin microbiota and related infections [21, 22].

*Micrococcus luteus* may cause bacteraemia and other conditions in immunocompromised patients [23, 24]. Notably, it can survive in nutrient-deprived conditions for years, which makes it similar to *E. faecalis*, a well-known endodontic pathogen [25]. Due to its survival properties and ability to form a biofilm, *M. luteus* appears to be naturally adapted for colonising the root canal system and supporting chronic periodontitis.

In our study, *Stenotrophomonas maltophilia* was frequently detected, particularly in DUWL samples but also across other clinical sources, underscoring its adaptability to diverse reservoirs within the dental setting. It is increasingly recognised as a cause of healthcare-associated infections due to its intrinsic multidrug resistance and ability to persist in water-related biofilms [26]. Its detection in dental unit waterlines highlights a potential risk of cross-contamination in dental practice, emphasising the need for strict monitoring and effective disinfection protocols [27, 28].

When summarising the relationship between the identified pathogens and their clinical implications, it is important to highlight the aetiological role of *S. epidermidis* in infective endocarditis (IE) [17, 29]. Its strains were frequently found and possessed antibiotic resistance, which is an aggravating factor. Along with frequently found *S. epidermidis* and *Micrococcus luteus*, a number of the bacteria found can cause bacteraemia, which is especially threatening to immunocompromised patients and patients with cardiac conditions. The presence of implants such as artificial heart valves, pacemakers, artificial joints, and others may pose an additional threat when nosocomial bacteria are introduced into bloodstream during endodontic treatment. Frequently found strains of *Stenotrophomonas maltophilia* may impose risk of various infections, especially those of respiratory tract.

Microbial colonies were detected on both the working and peripheral parts of gloves and rubber dam sheets. Contamination was present not only on the areas directly involved in manipulations but also on peripheral regions, such as near the patient’s nose or closer to the operator’s skin. This findings highlight that microbial transfer can occur to different regions of a single clinical item, emphasising the importance of considering all contact surfaces as potential reservoirs of infection. To the best of our knowledge, this is the first report drawing attention to these peripheral sites, and it supports the need to regularly disinfect such areas during treatment, or to replace the material before obturation [30].

No bacteria were found on gutta-percha points, possibly due to their smooth surface and the antimicrobial properties of ZnO, a primary component of gutta-percha points [31, 32]. We presume that a wider range of samples might show some detectable contamination, but it would still be less than that found on other surfaces tested in this research. This finding confirms the data provided by other authors [33, 34].

High microbial contamination was observed in DUWL samples. It could be explained as this is a particularly problematic niche for microbial contamination, as the narrow-bore tubing, humidity, reverse suction, insufficient disinfection, intermittent water flow, and nutrient-rich environment favour persistent biofilm formation. These biofilms can act as reservoirs for opportunistic and resistant microorganisms, posing a risk of nosocomial infection and creating challenges for disinfection in routine dental practice [15, 35, 36].

Contamination of the working areas of rubber dam sheets may result from repeated contact during clinical manipulations and saliva exposure, for example, during X-ray sensor positioning. Similarly, the near-the-skin parts of gloves may be prone to contamination due to the ubiquitous presence of bacteria on staff skin. These findings emphasise the importance of regular disinfection or replacement of these items during treatment.

Microorganisms were also isolated from other routinely used clinical items, such as paper points, endodontic dispensers, and files. Although the clinical role of each object differs, their contamination demonstrates that multiple potential reservoirs of nosocomial bacteria exist in the endodontic setting. Contaminated paper points may pose a particularly high risk of infection because they are introduced into the root canal at the end of disinfection procedures. In contrast, endodontic files are inserted with an irrigant, mostly sodium hypochlorite, which may reduce viable microbial transfer.

Frequent antibiotic resistance was observed among the tested strains. *Staphylococcus epidermidis*, with sufficient cases to determine statistical frequency, often exhibited multiresistance (see Table S1). Recent studies indicate that *S. epidermidis*, a predominant human skin dweller, carries diverse resistance determinants in both environmental and clinical strains, serving as an important reservoir of resistance genes [37, 38]. Moreover, *S. epidermidis* biofilm-associated infections—particularly those related to indwelling devices—are notoriously difficult to treat and often linked to recurrent infection or treatment failure [39].

The predominance of *S. epidermidis* and *M. luteus* on gloves and rubber-dam sheets likely reflects breaches in aseptic protocols. Notably, molecular-typing studies have demonstrated that *S. epidermidis* strains isolated from endodontic lesions represent true nosocomial pathogens—distinct from merely commensal skin flora—underlining their clinical relevance [16, 40, 41].

There is a regulation on drinking water called “Coli index”. In this research, the water from a dental unit bottle was examined, not from a general supply of drinking water. Considering that all objects in the patient’s vicinity must be sterile, the role of disinfection, sterilisation, and maintenance of instruments and devices is especially important. This is particularly relevant in dentistry, where strict asepsis may be less consistently implemented than in surgical departments.

In planning the number of samples, we aimed to replicate typical clinical conditions and protocols and ensure a balanced representation. However, it should be acknowledged that microbial counts from different types of samples (e.g., liquids versus surfaces) are not directly comparable, and the results are therefore presented descriptively rather than quantitatively. Variations in contamination levels may also exist between different types of clinics, such as teaching-oriented clinics with numerous students and patients compared to private practices with fewer patients per day. Given the limited amount of water in the collected DUWL samples, it appears difficult to identify, for instance, *Legionella*, which is an additional limitation of this study.

It should be acknowledged that our study was conducted in a single private dental setting, which ensures internal consistency but limits generalisability. Microbial contamination levels may vary between clinics due to differences in patient populations, staff, and specific protocols. Therefore, these results should be interpreted in the context of previous studies investigating microbial contamination in endodontic practice.

In order to reduce nosocomial endodontic infections, the following hygienic measures are recommended:

1. Avoid using DUWL water as an irrigant.
2. Sterilise paper points or use presterilised ones.
3. Use large rubber dam sheets to completely cover the patient’s nose and partially cover skin regions, providing sufficient coverage for the doctor’s hands beyond the minimal working area.
4. Decontaminate the rubber dam sheet immediately after placement and then regularly during the treatment, especially before the obturation stage.
5. Decontaminate gloves immediately after putting them on and regularly during treatment, especially after X-rays. Preferably, use a contactless antiseptic dispenser or change gloves before the obturation stage.
6. Use autoclaved glass mixing slabs instead of mixing pads.
7. Replace reusable endodontic dispensers with single-use syringes.

Future research could investigate the ability of the detected nosocomial infection to survive contact with freshly mixed sealers.

### Ethics statement

The study did not require ethical approval because it involved only microbiological and environmental sampling of clinical materials and did not include any intervention or experimental procedure involving patients. Informed consent was obtained from all subjects involved in the study.

## Conclusions

1. Mixing pads, paper points, water from a dental unit water line (DUWL), rubber dam sheets (working and near-the-nose parts), gloves (working and near-the-skin parts), endodontic dispensers (reusable syringes for irrigation), and endodontic files (stored in an open sponge after sterilisation) used in routine endodontic practice during patient treatment are contaminated with microorganisms.
2. *Staphylococcus epidermidis* and *Micrococcus luteus* are the prevalent species found on most objects routinely used in endodontics. *Stenotrophomonas maltophilia* is a prevalent species in DUWL.
3. Microbial contamination was observed across different categories of items and across different regions of the same item (e.g., gloves and rubber dam sheets), underlining that multiple surfaces can act as potential microbial reservoirs.
4. Gutta-percha points are free of culturable microorganisms.
5. Endodontic nosocomial bacteria frequently possess antibiotic resistance.

## Supplementary information

Supplementary Table S1

## Data Availability

The data sets obtained and analysed during this study are presented in the manuscript or can be made available from the corresponding author upon reasonable request.
