# Supplementary material for: Evaluation of potential sources of nosocomial infection in endodontic practice: a hygienic study
Source: BDJ Open. 2025 Dec 12;11:95. doi: 10.1038/s41405-025-00378-w (PMC12701015; doi:10.1038/s41405-025-00378-w)
Supplement: Supplementary file 1 — Supplementary Table S1 [file 41405_2025_378_MOESM1_ESM.docx]

Table S1. Antibiotic susceptibility of the most frequent bacterial isolates (N ≥ 3).

| Species (N) | Sources | Antibiotic | Resistant (R), n (%) | Intermediate (I), n (%) | Susceptible (S), n (%) |
| --- | --- | --- | --- | --- | --- |
| *S. epidermidis* (N=6) | DUWL, RDW, GW | Azithromycin | 3 (50%) | 0 (0%) | 3 (50%) |
|  |  | Trimethoprim | 1 (~17%) | 0 (0%) | 5 (~83%) |
|  |  | Clarithromycin | 3 (50%) | 0 (0%) | 3 (50%) |
|  |  | Penicillin | 3 (50%) | 0 (0%) | 3 (50%) |
|  |  | Oxacillin | 1 (~17%) | 0 (0%) | 5 (~83%) |
|  |  | Erythromycin | 3 (50%) | 0 (0%) | 3 (50%) |
|  |  | Linezolid | 0 (0%) | 0 (0%) | 6 (100%) |
|  |  | Tetracycline | 1 (~17%) | 0 (0%) | 5 (~83%) |
|  |  | Rifampin | 0 (0%) | 0 (0%) | 6 (100%) |
|  |  | Minocycline | 0 (0%) | 0 (0%) | 6 (100%) |
|  |  | Vancomycin | 0 (0%) | 0 (0%) | 6 (100%) |
|  |  | Daptomycin | 0 (0%) | 0 (0%) | 6 (100%) |
|  |  | Doxycycline  Note: 1 isolate not tested | 0 (0%) | 0 (0%) | 5 (100%) |
|  |  | Chloramphenicol | 0 (0%) | 0 (0%) | 6 (100%) |
|  |  | Ciprofloxacin | 0 (0%) | 0 (0%) | 6 (100%) |
|  |  | Levofloxacin | 0 (0%) | 0 (0%) | 6 (100%) |
|  |  | Moxifloxacin | 0 (0%) | 0 (0%) | 6 (100%) |
|  |  | Gentamicin | 0 (%) | 2 (~33%) | 4 (~67%) |
|  |  | Tobramycin | 0 (%) | 2 (~33%) | 4 (~67%) |
|  |  | Amikacin | 0 (0%) | 0 (0%) | 6 (100%) |
|  |  | Teicoplanin | 0 (0%) | 0 (0%) | 6 (100%) |
|  |  | Tigecycline | 0 (0%) | 0 (0%) | 6 (100%) |
|  |  | Clindamycin | 0 (0%) | 0 (0%) | 6 (100%) |
| *M. luteus*  (N=6) | RDW, GW | Penicillin | 1 (~17%) | 0 (0%) | 5 (~83%) |
|  |  | Erythromycin | 2 (~33%) | 1 (~17%) | 3 (50%) |
|  |  | Vancomycin | 1 (~17%) | 0 (0%) | 5 (~83%) |
|  |  | Clindamycin | 1 (~17%) | 0 (0%) | 5 (~83%) |
| *S. aureus*  (N=3) | RDN, GS | Azithromycin | 3 (100%) | 0 (0%) | 0 (0%) |
|  |  | Trimethoprim | 0 (0%) | 0 (0%) | 3 (100%) |
|  |  | Clarithromycin | 3 (100%) | 0 (0%) | 0 (0%) |
|  |  | Penicillin | 2 (~67%) | 0 (0%) | 1 (~33%) |
|  |  | Oxacillin | 0 (0%) | 0 (0%) | 3 (100%) |
|  |  | Erythromycin | 3 (100%) | 0 (0%) | 0 (0%) |
|  |  | Linezolid | 0 (0%) | 0 (0%) | 3 (100%) |
|  |  | Tetracycline | 0 (0%) | 1 (~33%) | 2 (~67%) |
|  |  | Rifampin | 0 (0%) | 0 (0%) | 3 (100%) |
|  |  | Minocycline | 0 (0%) | 0 (0%) | 3 (100%) |
|  |  | Vancomycin | 0 (0%) | 0 (0%) | 3 (100%) |
|  |  | Daptomycin | 0 (0%) | 0 (0%) | 3 (100%) |
|  |  | Doxycycline | 0 (0%) | 0 (0%) | 3 (100%) |
|  |  | Chloramphenicol | 1 (~33%) | 0 (0%) | 2 (~67%) |
|  |  | Ciprofloxacin | 0 (0%) | 0 (0%) | 3 (100%) |
|  |  | Levofloxacin | 0 (0%) | 0 (0%) | 3 (100%) |
|  |  | Moxifloxacin | 0 (0%) | 0 (0%) | 3 (100%) |
|  |  | Gentamicin | 0 (0%) | 0 (0%) | 3 (100%) |
|  |  | Tobramycin | 0 (0%) | 0 (0%) | 3 (100%) |
|  |  | Amikacin | 0 (0%) | 0 (0%) | 3 (100%) |
|  |  | Teicoplanin | 0 (0%) | 0 (0%) | 3 (100%) |
|  |  | Tigecycline | 0 (0%) | 0 (0%) | 3 (100%) |
|  |  | Cefotaxime | 3 (100%) | 0 (0%) | 0 (0%) |
|  |  | Clindamycin | 0 (0%) | 1 (~33%) | 2 (~67%) |

Note: N = total number of isolates of a given species tested against all listed antibiotics. n (%) = absolute number and percentage of isolates (out of N) showing resistance (R), intermediate resistance (I), or susceptibility (S). Percentages are calculated relative to N for each species. Only species isolated ≥3 times and tested against antibiotics are included. Sources: DUWL – dental unit water line, RDW – working region of rubber dam sheets, GW – working region of gloves, RDN – near-the-nose region of rubber dam sheets, GS – near-the-skin region of gloves.
